# Artificial intelligence–enabled sinus electrocardiograms for the detection of paroxysmal atrial fibrillation benchmarked against the CHARGE-AF score

**DOI:** 10.1093/ehjdh/ztaf100

**Published:** 2025-08-22

**Authors:** Constantine Tarabanis, Vidya Koesmahargyo, Dimitrios Tachmatzidis, Vasileios Sousonis, Constantinos Bakogiannis, Robert Ronan, Scott A Bernstein, Chirag Barbhaiya, David S Park, Douglas S Holmes, Alexander Kushnir, Felix Yang, Anthony Aizer, Larry A Chinitz, Stylianos Tzeis, Vassilios Vassilikos, Lior Jankelson

**Affiliations:** Division of Cardiovascular Medicine, Department of Medicine, Brigham and Women’s Hospital, Harvard Medical School, Boston, MA, USA; Leon H. Charney Division of Cardiology, NYU Langone Health, New York University School of Medicine, New York, NY 10016, USA; 3rd Cardiology Department, Hippokrateion University Hospital, Aristotle University of Thessaloniki, Thessaloniki, Greece; Department of Cardiology, Mitera Hospital Hygeia Group, Athens, Greece; 3rd Cardiology Department, Hippokrateion University Hospital, Aristotle University of Thessaloniki, Thessaloniki, Greece; Leon H. Charney Division of Cardiology, NYU Langone Health, New York University School of Medicine, New York, NY 10016, USA; Leon H. Charney Division of Cardiology, NYU Langone Health, New York University School of Medicine, New York, NY 10016, USA; Leon H. Charney Division of Cardiology, NYU Langone Health, New York University School of Medicine, New York, NY 10016, USA; Leon H. Charney Division of Cardiology, NYU Langone Health, New York University School of Medicine, New York, NY 10016, USA; Leon H. Charney Division of Cardiology, NYU Langone Health, New York University School of Medicine, New York, NY 10016, USA; Leon H. Charney Division of Cardiology, NYU Langone Health, New York University School of Medicine, New York, NY 10016, USA; Leon H. Charney Division of Cardiology, NYU Langone Health, New York University School of Medicine, New York, NY 10016, USA; Leon H. Charney Division of Cardiology, NYU Langone Health, New York University School of Medicine, New York, NY 10016, USA; Leon H. Charney Division of Cardiology, NYU Langone Health, New York University School of Medicine, New York, NY 10016, USA; Department of Cardiology, Mitera Hospital Hygeia Group, Athens, Greece; 3rd Cardiology Department, Hippokrateion University Hospital, Aristotle University of Thessaloniki, Thessaloniki, Greece; Leon H. Charney Division of Cardiology, NYU Langone Health, New York University School of Medicine, New York, NY 10016, USA

**Keywords:** Atrial fibrillation, Artificial intelligence, Electrocardiogram, CHARGE-AF score

## Abstract

**Aims:**

We aimed to develop and externally validate a convolutional neural network (CNN) using sinus rhythm electrocardiograms (ECGs) and CHARGE-AF features to predict incident paroxysmal atrial fibrillation (AF), benchmarking its performance against the CHARGE-AF score.

**Methods and results:**

We curated 157 192 sinus ECGs from 76 986 patients within the New York University (NYU) Langone Health system, splitting data into training, validation, and test sets. Two cohorts, from suburban US outpatient practices and Greek tertiary hospitals, were used for external validation. The model utilizing the sinus ECG signal and all CHARGE-AF features achieved the highest test set area under the receiver operator curve (AUC) (0.89) and area under the precision recall curve (AUPRC) (0.69), outperforming the CHARGE-AF score alone. Model robustness was maintained in the external US cohort (AUC 0.90, AUPRC 0.67) and the European cohort (AUC 0.85, AUPRC 0.78). Subgroup analyses confirmed consistent performance across age, sex, and race strata. A CNN using ECG signals alone retained strong predictive ability, particularly when simulating missing or inaccurate clinical data.

**Conclusion:**

Our CNN integrating sinus rhythm ECGs and CHARGE-AF features demonstrated superior predictive performance over traditional risk scoring alone for detecting incident paroxysmal AF. The model maintained accuracy across geographically and clinically diverse external validation cohorts, supporting its potential for broad implementation in AF screening strategies.

## Graphical Abstract

**Graphical Abstract:**
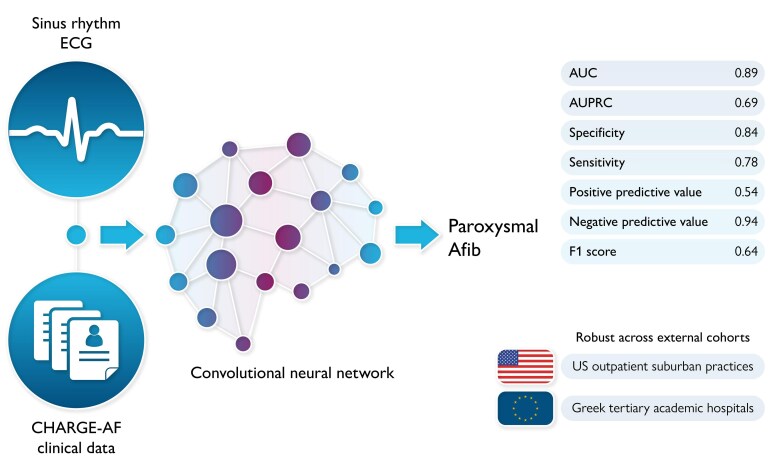

## Introduction

Atrial fibrillation (AF), characterized by the irregular beating of the atrial chambers, is the most common sustained cardiac arrhythmia^1^ and is a major cause of stroke, heart failure, and sudden death.^2^ Though key to preventing subsequent complications, timely AF diagnosis is challenging. Up to 20% of AF patients are asymptomatic (‘silent’), while another one-third have atypical or non-specific symptoms.^3^ The remaining cases present as infrequent symptomatic episodes before progressing, years later, to longer and more frequent attacks.^2^ An electrocardiogram (ECG) would have to be obtained at the time of arrhythmia and if not, a normal sinus rhythm (NSR) ECG provides false reassurance.

Consequently, AF often remains undiagnosed,^4^ which is especially alarming considering ischaemic stroke can be its first presentation.^5^ Characteristically, 15% of patients with an embolic stroke of undetermined source undergoing rhythm monitoring were found to have previously undiagnosed AF.^6^ The detection of AF is pivotal for the early initiation of management strategies to mitigate its impact. For example, prompt initiation of oral anticoagulation can lower stroke risk,^7^ whereas long-term post-ablation success rates are highest in early paroxysmal AF.^8^

To inform targeted screening, neural networks utilizing the sinus ECG signal as their primary input (AI-ECG) have been developed for the detection of paroxysmal AF in retrospective cohorts.^9–12^ However, there are conflicting reports on the additive AF predictive value of such AI-ECG models against established clinical risk scores such as the CHARGE-AF^13^ score, illustrating either equivalent^10,11^ or only marginally greater^12^ predictive power. We report an ECG-based convolutional neural network (CNN) for the detection of incident AF illustrating its superior performance and clinical utility against the CHARGE-AF score and externally validating it in both local suburban outpatient practices and European tertiary referral hospitals.

## Methods

### Cohort selection

**Figure 1:**
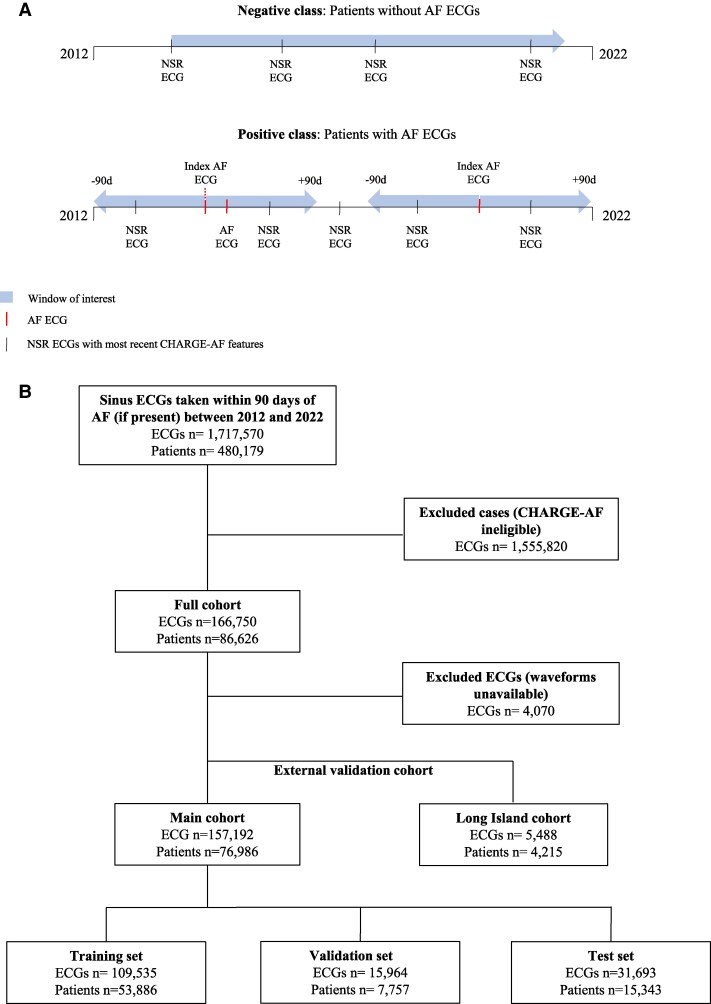
Study design. (*A*) Diagram illustrating the study design. For the purposes of model development, patients with no documented history of atrial fibrillation were considered the negative class, whereas patients with at least one MUSE-labelled atrial fibrillation electrocardiogram were considered the positive class. For the negative class, all identified normal sinus rhythm electrocardiograms and for the positive class normal sinus rhythm electrocardiograms within ±90 days of an atrial fibrillation electrocardiogram were used as inputs for model training. (*B*) The study design flow chart for identifying normal sinus rhythm electrocardiograms with recently documented CHARGE-AF features in our institutional database and for defining the training, validation, test (7:1:2), and external validation sets. AF, atrial fibrillation; ECG, electrocardiogram; NSR, normal sinus rhythm.

We identified all patients with 12-lead ECGs obtained within the New York University (NYU) Langone Health system between 1 January 2012 and 1 January 2022. The cohort included two groups of patients based on the presence or absence of a documented history of AF ascertained by ECG labels generated by MUSE (GE Healthcare, Chicago, IL), which are reviewed and confirmed or overread by a cardiologist as part of a standard clinical workflow at our institution. For patients with no AF ECG recorded, all NSR ECGs were identified and included. For patients with at least one AF ECG recorded, all NSR ECGs obtained within ±90 days of an AF ECG were identified and included. If multiple AF ECGs were obtained within 180 days of each other, the earliest (index) AF ECG occurrence was used to identify NSR ECGs for inclusion (*Figure 1A*). Patients who had no record of an AF ECG in the electronic health record (EHR), but otherwise had a documented history of an AF diagnosis, were excluded from the study population. The resulting study cohort of NSR ECGs belonging to patients with and without a history of AF was split into training, validation, and test sets in a 7:1:2 ratio (*Figure 1B*), and their characteristics are listed in Supplementary material online, *Table S1*. The ECGs obtained at NYU Long Island served as an external validation cohort and were not included in the main cohort (*Figure 1B*; Supplementary material online, *Table S2*). A second external validation cohort (see Supplementary material online, *Table S3*) was derived from Mitera Hospital, a private hospital located in Athens, Greece, and Ippokrateio General Hospital, a public hospital affiliated with the Aristotle University of Thessaloniki (AUTH), also located in Greece. This European external validation cohort included NSR ECGs at any time point in relation to an AF episode. Both the NYU Langone Health Institutional Review Board and Independent Ethics Committee for each hospital in Greece approved the study protocol and waived the need for informed consent. The study complied with the principles of the Declaration of Helsinki. Finally, the present study and manuscript were designed and composed in alignment with the European Heart Rhythm Association’s artificial intelligence (AI) checklist for reporting, reading, and understanding AI studies in clinical electrophysiology (see Supplementary material online, *Table S4*).^14^

### Model inputs

Encounter data were collected from the NYU Langone Health EHR (Epic Systems, Verona, WI), and ECGs were retrieved from MUSE as XML files (GE Healthcare, Chicago, IL). The CHARGE-AF score was calculated using clinical features that preceded each ECG acquisition date. If CHARGE-AF features were missing from the EHR, then these cases were excluded. The CHARGE-AF score was calculated as previously described^13^:


$$1-0.9718412736^{\exp(\sum \beta X-12.5815600)}$$

where ∑βX denotes the linear combination of the following covariates and their respective published β-coefficients: past medical history (diabetes, myocardial infarction, heart failure), demographics (age, race, current smoking), vitals (height, weight, systolic/diastolic blood pressure), and use of anti-hypertensive medication.

A total of five models were developed as described below to detect incident AF from NSR ECGs: ECG + CHARGE-AF, ECG + past medical history (PMH), ECG + demographics, ECG + vitals, and ECG only. As suggested by their names, the models differed in the type of inputs used for model development, which included the NSR ECG time series and tabular data corresponding to all or subsets (as described above) of the CHARGE-AF score’s features. They were compared with the CHARGE-AF score and a CHARGE-AF model that used the features as inputs into a CNN. Whenever more than one NSR ECG was available and eligible for inclusion as determined by the study design (*Figure 1A*), then the time series for all were utilized as model inputs during training.

### Model architecture

A CNN was implemented to learn a concise one-dimensional representation of the NSR ECG time series.^15^ This representation was concatenated with the tabular data then fed through a fully connected neural network with a softmax output layer to generate the probability of each class. The CNN architecture was based on the current state of the art for arrhythmia detection^16^ and is a 34-layer ResNet CNN consisting of 16 residual connections as depicted in Supplementary material online, *Figure S1*. The input to the network was an 8 × 2500 matrix representing eight measured leads (I, II, V1–6) by 10-s duration sampled at 250 Hz (500 Hz ECGs were downsampled to 250 Hz).

### Model training and hyperparameter tuning

The aforementioned data inputs were used to train the models in detecting the presence of paroxysmal AF. The models were trained using an Adam optimizer for 100 epochs to minimize the categorical cross-entropy loss. After each epoch, the models were evaluated on the internal validation set. A learning rate scheduler was used that multiplies the learning rate by 0.8 after two epochs of no validation loss improvement. Early stopping was triggered after the validation loss ceased to improve for five epochs. We used the validation set to select the best network architecture and hyperparameter configuration. We selected the model with the highest average area under the precision recall curve (AUPRC).

### Model evaluation

Both the test and external validation sets were used to evaluate model performance in detecting incident AF. Based on Youden’s index,^17^ thresholds of 0.21 and 0.02 were used to derive the model performance metrics for all CNN models and the CHARGE-AF score, respectively. The receiver operator and precision recall curves were constructed and the area under each was calculated. We then calculated model thresholds that correspond to commonly accepted true positive rate (TPR) values to classify the model output. The positive predictive value (PPV) at each TPR was assessed for all models.

### Statistics

Values are presented as median and interquartile range (IQR) for continuous variables and as count (percentage) for categorical variables, unless otherwise specified. Analysis of variance was used for comparisons of continuous variables among >2 groups, while the χ^2^ test was used for comparisons of categorical variables. The two-sample Kolmogorov–Smirnov test was applied to assess whether continuous variables (CHARGE-AF score) in two groups followed the same underlying distribution by comparing their empirical cumulative distribution functions. Statistical analysis was performed using the software Scikit-learn (version 1.02). Values of *P* < 0.05 were considered statistically significant.

## Results

The main cohort consisted of 157 192 NSR, 12-lead ECGs belonging to 76 986 patients (*Figure 1B*). The test set and external validation cohorts were used to evaluate model performance. The test set consisted of 31 693 NSR ECGs belonging to 15 343 unique patients of which 3064 (20%) had AF (see Supplementary material online, *Table S1*). Patients in the test set had a median age of 64 years (IQR: 53–74 years), had both sexes equally presented (female 49.2%) and were predominantly white (61.9%). Comorbidities pertaining to the CHARGE-AF score were also collected: current smoking (7.9%), diabetes (15.9%), heart failure (14.6%), hypertension (24.0%), and history of myocardial infarction (11.8%). Patients had a median systolic and diastolic blood pressure of 125 (IQR: 113–139) and 73 (IQR: 65–80) mm Hg, respectively, with 13.2% of patients on anti-hypertensive medications (see Supplementary material online, *Table S1*).

**Figure 2:**
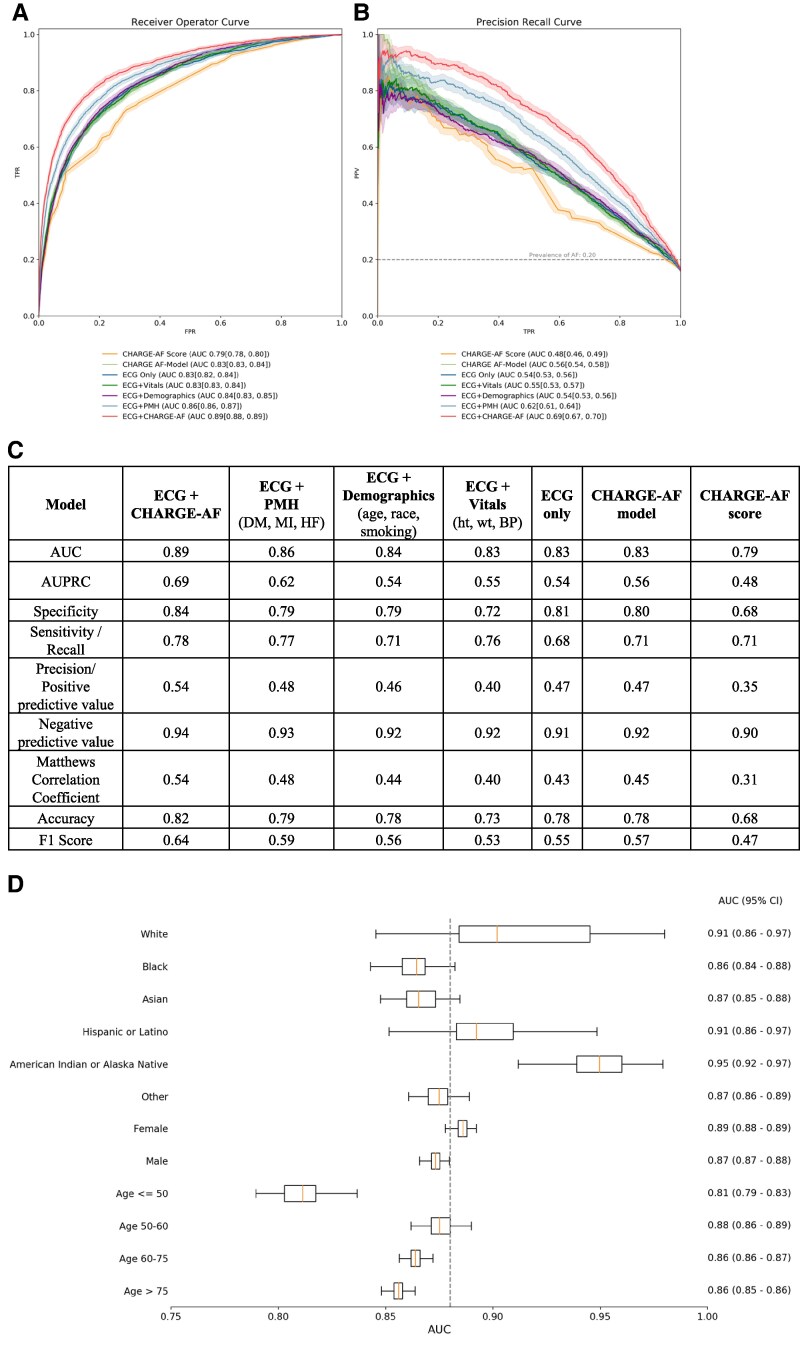
Main cohort test set. The receiver operator characteristic (*A*) and precision recall (*B*) curves for each convolutional neural network model applied to the test set. (*C*) A comprehensive set of model performance metrics for each convolutional neural network model applied to the test set. (*D*) The area under the receiver operator curve for the electrocardiogram + CHARGE-AF model in subgroups of race, sex, and age presented as a box (interquartile range) and whisker (95% confidence interval) plot. The dotted line represents the model’s area under the receiver operator curve for the test set in its entirety. AUC, area under the receiver operator curve; AUPRC, area under the precision recall curve; BP, blood pressure; CI, confidence interval; CNN, convolutional neural network; DM, diabetes mellitus; ECG, electrocardiogram; FPR, false positive rate; HF, heart failure; Ht, height; MI, myocardial infarction; PMH, past medical history; TPR, true positive rate; Wt, weight.

Six CNN-based models were developed as described in the Methods section and compared with the CHARGE-AF score. Their receiver operator and precision recall curves are depicted in *Figure 2A* and *B*, respectively. All models outperformed the CHARGE-AF score. The CNN model utilizing both the NSR ECG time series and all CHARGE-AF features achieved the highest area under the receiver operator curve (AUC) of 0.89 (95% CI: 0.88–0.89) and AUPRC of 0.69 (95% CI: 0.67–0.70). The addition of the past medical history CHARGE-AF features (diabetes, myocardial infarction, heart failure) to the model input afforded a greater predictive performance increase compared with adding the demographic (age, race, smoking) and vital sign (height, weight, blood pressure) components of the CHARGE-AF score (*Figure 2A* and *B*). With regard to model performance metrics (*Figure 2C*), the ECG + CHARGE-AF model achieved a specificity of 0.84, a sensitivity of 0.78, an accuracy of 0.82, an F1 score of 0.64, and negative and positive predictive values of 0.94 and 0.54, respectively (*Figure 2C*). It outperformed all other models’ metrics, including the CHARGE-AF score. The ECG + CHARGE-AF model’s AUCs for subgroups of race, sex, and age are presented as a box-and-whisker plot in *Figure 2D*. The best performance in each subgroup is noted among patients of self-reported American Indian or Alaska Native descent (AUC 0.95, 95% CI: 0.92–0.97), female sex (AUC 0.89, 95% CI: 0.88–0.89), and aged 50–60 years (AUC 0.88, 95% CI: 0.86–0.89).

In order to investigate the models’ performance in cases of inaccurate and/or missing EHR entries, a subset of CHARGE-AF features of patients in the test set were then randomly permuted, while the ECG waveform in each sample remained intact. In this case, the CNN model utilizing the ECG time series alone achieved the highest AUC of 0.83 (95% CI: 0.83–0.84) and AUPRC of 0.54 (95% CI: 0.64–0.70; Supplementary material online, *Figure S2B*). With regard to model performance metrics (see Supplementary material online, *Figure S2C*), the ECG-only model achieved a specificity of 0.81, a sensitivity of 0.68, an accuracy of 0.78, an F1 score of 0.55, and negative and positive predictive values of 0.91 and 0.47, respectively, outperforming most other models’ metrics.

**Figure 3:**
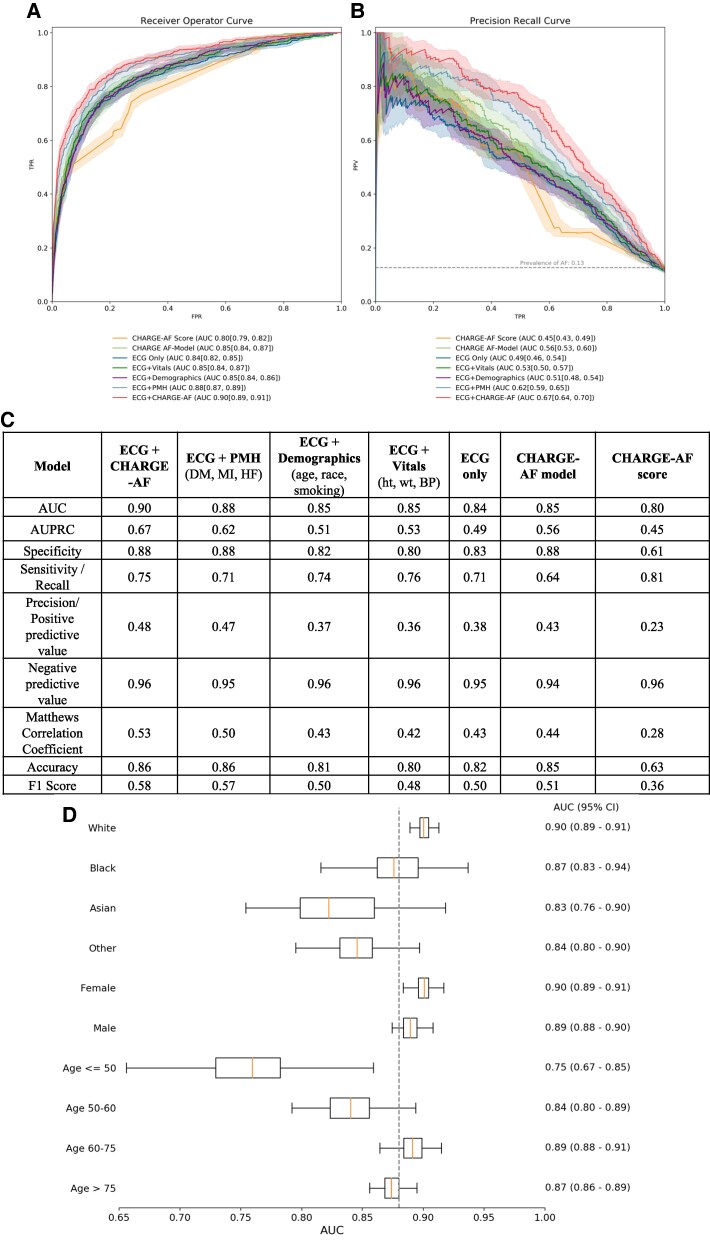
US external validation. The receiver operator characteristic (*A*) and precision recall (*B*) curves for each convolutional neural network model applied to the local external validation cohort. (*C*) A comprehensive set of model performance metrics for each convolutional neural network model applied to the local external validation cohort. (*D*) The area under the receiver operator curve for the electrocardiogram + CHARGE-AF model in subgroups of race, sex, and age presented as a box (interquartile range) and whisker (95% confidence interval) plot. The dotted line represents the model’s area under the receiver operator curve for the test set in its entirety. Abbreviations are as in *Figure 2*.

A local external validation cohort comprising local suburban outpatient practices consisted of 5488 NSR ECGs corresponding to 4215 patients, with a median age of 66 years (IQR: 56–76), who were predominantly female (53.2%) and white (75.6%; Supplementary material online, *Table S2*). Compared with the test set, a smaller proportion of patients in the local external validation cohort were current smokers (5.3%) and had diabetes (8.2%), heart failure (5.9%), or a history of myocardial infarction (1.6%). The same six CNN-based models were evaluated using the local external validation cohort as well and compared with the CHARGE-AF score (*Figure 3A* and *B*). Similar to the test set, the CNN model utilizing both the NSR ECG time series and all CHARGE-AF features achieved the highest AUC of 0.90 (95% CI: 0.89–0.91) and AUPRC of 0.67 (95% CI: 0.64–0.70). With regard to model performance metrics (*Figure 3C*), the ECG + CHARGE-AF model achieved a specificity of 0.88, a sensitivity of 0.75, an accuracy of 0.86, an F1 score of 0.58, and negative and positive predictive values of 0.96 and 0.48, respectively (*Figure 3C*). It outperformed all other models’ metrics, including the CHARGE-AF score. The ECG + CHARGE-AF model’s AUC for external validation cohort subgroups of race, sex, and age are presented as a box-and-whisker plot in *Figure 3D*. The best performance in each subgroup is noted among patients who were white (AUC 0.90, 95% CI: 0.89–0.91), female (AUC 0.90, 95% CI: 0.89–0.91), and aged 60–75 years (AUC 0.89, 95% CI: 0.88–0.91).

**Figure 4:**
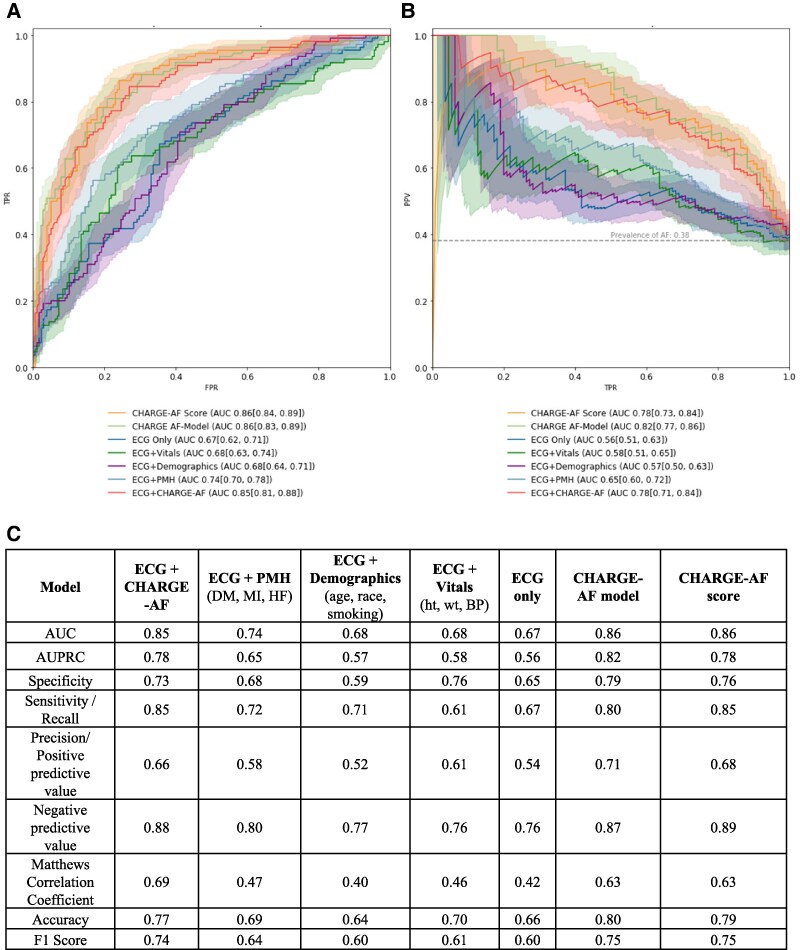
European external validation. The receiver operator characteristic (*A*) and precision recall (*B*) curves for each convolutional neural network model applied to the European external validation cohort. (*C*) A comprehensive set of model performance metrics for each convolutional neural network model applied to the European external validation cohort. (*D*) The area under the receiver operator curve for the electrocardiogram + CHARGE-AF model in subgroups of race, sex, and age presented as a box (interquartile range) and whisker (95% confidence interval) plot. The dotted line represents the model’s area under the receiver operator curve for the test set in its entirety. Abbreviations are as in *Figure 2*.

A European external validation cohort comprising patients from both private and public hospitals in Greece consisted of 306 NSR ECGs corresponding to 306 patients, with a median age of 57 years (IQR: 42–70), who were predominantly female (66%) and white (99%; Supplementary material online, *Table S3*). Compared with the test set, a greater proportion of patients were current smokers (31%) and had a history of myocardial infarction (2.9%), while a smaller proportion of patients had diabetes (12.7%) and heart failure (6.9%). The same six CNN-based models were evaluated using the European external validation cohort as well and compared with the CHARGE-AF score (*Figure 4A* and *B*). Unlike the test set and local external validation cohorts, the CNN model utilizing both the NSR ECG time series and all CHARGE-AF features achieved similar predictive performance metrics to CHARGE-AF model. In this external validation cohort, the ECG + CHARGE-AF achieved an AUC of 0.85 (95% CI: 0.81–0.88), an AUPRC of 0.78 (95% CI: 0.71–0.84), a specificity of 0.73, a sensitivity of 0.85, an accuracy of 0.77, an F1 score of 0.74, and negative and positive predictive values of 0.88 and 0.66, respectively (*Figure 4C*).

**Figure 5:**
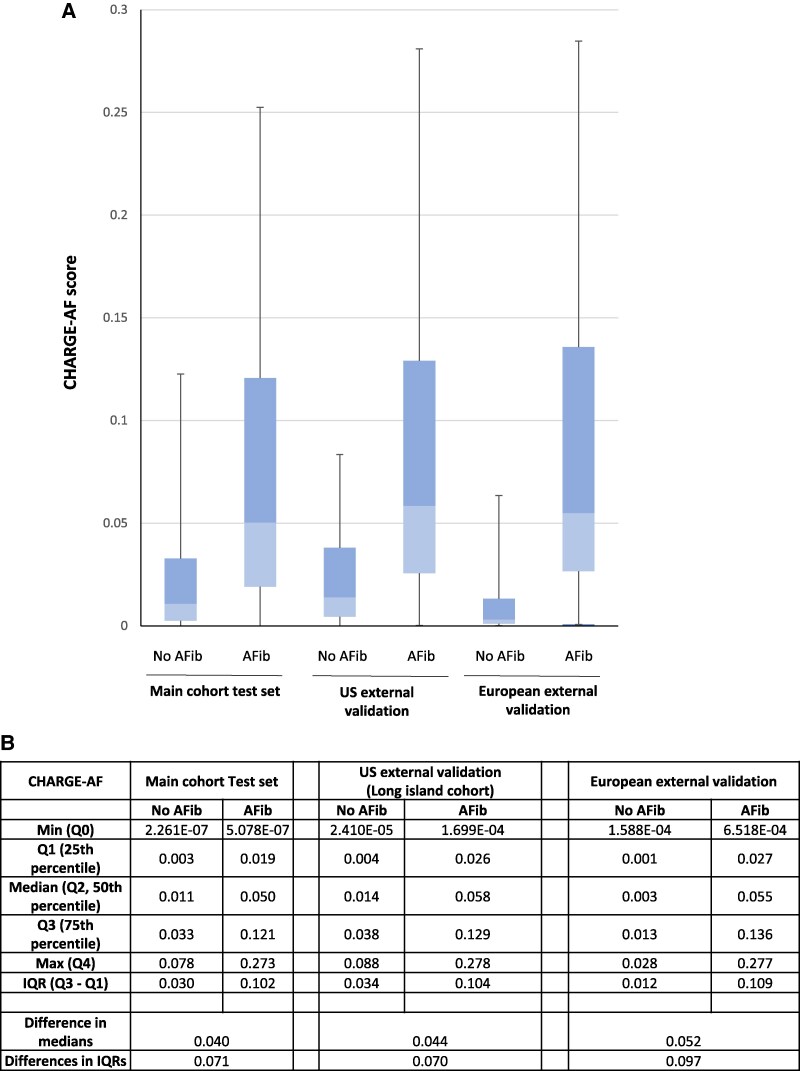
CHARGE-AF distribution analysis. (*A*) The CHARGE-AF score distributions for positive (atrial fibrillation) and negative (no atrial fibrillation) classes in the main cohort test set, local, and European external validation cohorts presented as box (interquartile range) and whisker (95% confidence interval) plots. (*B*) The corresponding CHARGE-AF score quartiles (Q0, Q1, Q2, Q3, Q4) and interquartile range for positive (atrial fibrillation) and negative (no atrial fibrillation) classes in the main cohort test set, local, and European external validation cohorts. AF, atrial fibrillation.

The CHARGE-AF scores were compared between patients with and without AF across the main cohort test set, local external validation cohort, and European external validation cohort (*Figure 5A*). In all cohorts, the median CHARGE-AF scores were higher in patients who developed AF than in those who did not. The IQR of CHARGE-AF scores was also wider in AF patients, reflecting greater variability in risk estimation (*Figure 5B*). The differences in median CHARGE-AF scores between AF and non-AF patients were 0.040, 0.044, and 0.052 in the main test set, local external validation cohort, and European external validation cohort, respectively. A two-sample Kolmogorov–Smirnov test comparing CHARGE-AF score distributions between the main cohort and the European external validation cohort yielded a KS statistic of 0.198 (*P* = 3.19 × 10^−10^), indicating a significant difference in the underlying distributions of CHARGE-AF scores between these cohorts.

## Discussion

In recent years, research has illustrated the ability of deep learning model architectures such as CNNs to detect the presence of incident paroxysmal AF using the sinus rhythm ECG signal as their primary input.^9–12^ Despite the novelty of these findings, key questions remain about the real-world clinical value of such models. Firstly, the clinical utility of AI-ECG models would be most apparent if their predictive performance surpassed that of existing clinical risk scores relying solely on tabular data, such as the CHARGE-AF score. Most prior published work in this field avoids such direct comparisons with a few notable exceptions showing conflicting results^10–12^ regarding the additive AF predictive value of AI-ECG models against the CHARGE-AF score. Secondly, most prior investigations^9,10,18^ lack an external validation cohort despite ongoing concerns about CNN overfitting after training on single institution ECG repositories with documented drops in AF AI-ECG model performance in certain clinically distinct populations.^12,19^ Lastly, prior studies lack certain key performance metrics such as AUPRC and PPV/NPV.^9–12,18,20^ In the highly imbalanced (positive << negative classes) datasets used to train and test AI-ECG models for incident AF prediction, algorithms designed to always predict the absence of AF would achieve a misleadingly high AUC, but not a high AUPRC. Hence, the AUPRC can be a more representative performance metric in this case.^21^

Additionally, it is important to clarify that our model was not trained to predict the ‘onset’ of AF, but rather to detect the presence of any recent paroxysmal AF episode (*Figure 1A*). This decision was driven by the challenges inherent to defining AF onset from both clinical and data capture standpoints. Clinically, a single, ‘true’ onset to AF cannot be defined, as many patients are asymptomatic or exhibit non-specific symptoms^3^ resulting in AF going undetected, which in turn is the very motivation for designing an AF AI-ECG model. Logistically, AF may have been recorded in settings not captured within the EHR, such as an outside emergency department. These factors render the true timing of AF onset uncertain for any AI-ECG model study design. Consequently, instead of linking predictions to a presumed onset, we defined a ±90-day window around each documented paroxysmal AF ECG and used associated sinus rhythm ECGs within that 180-day period for model training (*Figure 1A*). This framework better reflects real-world clinical scenarios in which patients may unknowingly harbour paroxysmal AF at the time of initial evaluation when an AF AI-ECG model would be analysing their sinus rhythm ECG.

Our study adds substantively to previous work by presenting a comprehensive performance evaluation of deep learning AI-ECG models for paroxysmal AF prediction including both local and European external validation cohorts, benchmarking against the CHARGE-AF score while investigating the relative contributions of CHARGE-AF features and the sinus ECG signal. We report the development of a CNN that utilizes the sinus rhythm ECG signal and CHARGE-AF score for the prediction of incident paroxysmal AF (*Figure 2A–C*), outperforming previously published models^11,12,16,18,19^ and maintaining robust AUPRC, PPV, and sensitivity at various clinical settings in the USA (*Figure 3A–C*) and abroad (*Figure 4A–C*). The ECG + CHARGE-AF model also demonstrated strong calibration, with predicted probabilities closely matching observed AF prevalence across risk strata (see Supplementary material online, *Figure S3*). This was further supported by a Brier score of 0.174, indicating reasonable overall accuracy in probabilistic prediction. With the sequential addition of risk factor subsets that comprise the CHARGE-AF score, we illustrate their relative contribution to AF prediction, but also the additive benefit of including the sinus rhythm ECG signal to the CHARGE-AF features for achieving improved AF predictions (*Figures 2A*–*C* and *3A–C*). The sequential addition of CHARGE-AF features by medical information type provided a degree of model interpretability, allowing insight into the relative predictive value of each feature group. More specifically, the greatest relative improvement in performance metrics (AUC, AUPRC, specificity, PPV, F1 score) was observed with the addition of past medical history features, whereas the separate addition of demographic and vital sign components yielded comparatively smaller gains (*Figures 2C, 3C,* and *4C*).

The external validation analyses demonstrated the models’ generalizability across various contexts. First, they confirmed geographic generalizability, as the models performed comparably in local suburban outpatient practices (*Figure 3A–C*) and in both private and public hospitals in a foreign country (*Figure 4A–C*)—settings distinct from the training dataset, which was derived from an urban academic hospital. Notably, each institution used different ECG machines from one another and from our home institution. Second, they provided evidence of clinical generalizability, given the distinct European patient population, which included a younger cohort with a higher prevalence of active smokers and prior MI but fewer cases of diabetes and heart failure (see Supplementary material online, *Table S3*). Additionally, subgroup analyses within both the test and local external validation cohorts further supported generalizability across patients with diverse demographic characteristics (*Figures 2D* and *3D*). Third, the results reinforced the models’ temporal generalizability. Unlike, the main cohort, sinus rhythm ECGs in the European external validation cohort were not necessarily recorded within 90 days of an AF ECG. Hence, in this case, the models were identifying paroxysmal AF from sinus rhythm ECGs obtained at any time relative to an AF event.

Among the developed models, the CNN utilizing only the ECG signal as input, without CHARGE-AF features, may offer distinct clinical value in appropriate settings. The ECG is a widely accessible, standardized, and reproducible data source, independent of geographic location.^22^ Thus, AI-ECG models that rely solely on the ECG signal for phenotypic predictions have clear utility in healthcare systems without an EHR. Furthermore, the computational demands of locally hosting neural networks for ECG analysis are significantly lower than those required to install and maintain an EHR, both in terms of infrastructure and human resources. Even in healthcare systems with EHRs, data quality can be poor, with missing or erroneous entries.^23,24^ To illustrate the potential advantages of using the ECG signal alone, we simulated a poorly curated EHR by randomly permuting CHARGE-AF features, demonstrating the robustness of ECG-based models in such scenarios (see Supplementary material online, *Figure S2A*–*C*). As expected, permutation of the CHARGE-AF features had no impact on the performance of the ECG-only model, whereas models based exclusively on CHARGE-AF features showed markedly diminished performance. Notably, the CHARGE-AF + ECG model, while still outperforming the CHARGE-AF model alone, underperformed compared with the ECG-only model (*Figure 2C* vs. Supplementary material online, *Figure S2C*). This pattern suggests a potential interaction effect between the ECG signal and feature-based inputs within the neural network architecture, offering further insight into the model’s internal representations and interpretability.

In healthcare systems that maintain large-scale and well-curated EHRs, AI-ECG models would have to illustrate clear predictive superiority over traditional risk factor models to ensure distinct clinical utility. In the case of paroxysmal AF, a significant additive predictive value of the ECG signal over the CHARGE-AF score has not been consistently illustrated in the literature to date.^10–12^ In our study, the addition of the ECG signal to the CHARGE-AF as model inputs improved paroxysmal AF predictions in an urban academic hospital (*Figure 2A–C*) and a local external validation cohort of suburban outpatient practices (*Figure 3A–C*). The incremental improvement achieved by incorporating the ECG signal suggests that the CNN captures a ‘concealed’ risk signal embedded in the ECG. This may be due to the AI model identifying known risk factors that are inaccurately or insufficiently documented in the EHR or detecting previously unknown risk features within the signal.^25^

Unlike the testing set and local external validation cohort, the European external validation cohort included only hundreds of patients rather than thousands. This substantially smaller sample size rendered this analysis more susceptible to sampling bias, as evidenced by the overperformance of the CHARGE-AF score in this cohort (AUC 0.86; *Figure 4A* and *C*) compared with its previously published range (AUC 0.60–0.80).^26^ This bias is illustrated by the CHARGE-AF score distributions among patients with and without AF, which showed a significantly greater separation between positive and negative classes in the European cohort compared with the testing dataset (*P* = 3.19 × 10^−10^; *Figure 5A* and *B*). These findings underscore a key insight regarding the additive value of the ECG signal over established clinical risk scores: when a subsample happens to have a greater separation in CHARGE-AF scores between positive and negative classes, the incremental predictive contribution of the ECG signal may be minimized and vice versa. This phenomenon likely contributes to the wide range of reported CHARGE-AF AUCs in the literature^26^ and the inconsistent findings regarding the added value of clinical scores vs. ECG signal in AI-ECG models for AF prediction.^10–12^ To ensure meaningful comparisons and interpretability, future publications on AI-ECG models for AF prediction should report CHARGE-AF score distributions within their study populations.

Delving deeper into the models’ performance in the European external validation cohort, one could notice the comparative underperformance of the ECG-only CNN model from an AUC standpoint. The AUC can appear misleadingly high in imbalanced datasets that are dominated by negative classes.^21^ Conversely, when comparing across datasets with differing class imbalances, the AUC can misleadingly suggest underperformance in cohorts with higher positive class prevalence. In contrast, metrics such as PPV and F1 score, which account for both sensitivity and precision, and AUPRC^21^ are more informative in such scenarios. The European external validation cohort had the highest AF prevalence (41%) compared with the test (29%) and local external validation cohorts (15.9%; Supplementary material online, *Tables S1*–*S3*). Consequently, in the case of the European external validation cohort, the ECG-only CNN model demonstrated a lower AUC (0.67; *Figure 2C*) compared with the test (0.83; *Figure 3C*) and local external validation (0.84; *Figure 4C*) sets. However, its AUPRC (0.56), PPV/precision (0.54), and F1 score (0.60) were all higher than the corresponding ECG-only CNNs in the other cohorts. These metrics better reflect the model’s clinical usefulness in high-prevalence populations, where accurate identification of true positives is particularly critical. In other words, the ECG-only CNN is making more correct positive predictions in this setting, aligning with the observed increase in AF prevalence.

This study has additional limitations, including its retrospective, single-centre design. However, the inclusion of subgroup analyses and two separate external validation cohorts helps mitigate concerns about generalizability. Additionally, implementing this AI model in clinical practice requires healthcare facilities to have an EHR system and a digital ECG database to extract relevant risk factors and raw ECG signals for CNN-based analysis. Future research will focus on validating the CHARGE-AF + ECG model prospectively, which is currently underway in a clinical trial setting.

## Conclusions

We present a CNN-based AI-ECG model that integrates the sinus rhythm ECG signal and CHARGE-AF score for the prediction of incident paroxysmal AF. Our model demonstrates superior predictive performance compared with the CHARGE-AF score alone, with a robust array of performance metrics (AUC, AUPRC, sensitivity, specificity, PPV/NPV, F1 score) across diverse clinical settings. Notably, its performance was maintained in external validation cohorts, including both local suburban outpatient practices and European tertiary care hospitals. These findings support the potential utility of AI-ECG models for AF screening beyond traditional risk scores.

## Supplementary Material

ztaf100_Supplementary_Data

## Data Availability

The data underlying this article will be shared on reasonable request to the corresponding author.
