# Supplementary material for: Artificial intelligence–enabled sinus electrocardiograms for the detection of paroxysmal atrial fibrillation benchmarked against the CHARGE-AF score
Source: Eur Heart J Digit Health. 2025 Aug 22;6(6):1134–44. doi: 10.1093/ehjdh/ztaf100 (PMC12629645; doi:10.1093/ehjdh/ztaf100)
Supplement: ztaf100_Supplementary_Data [file ztaf100_supplementary_data.zip › AFib Supp Figures 7.10.pptx]

## Slide 1
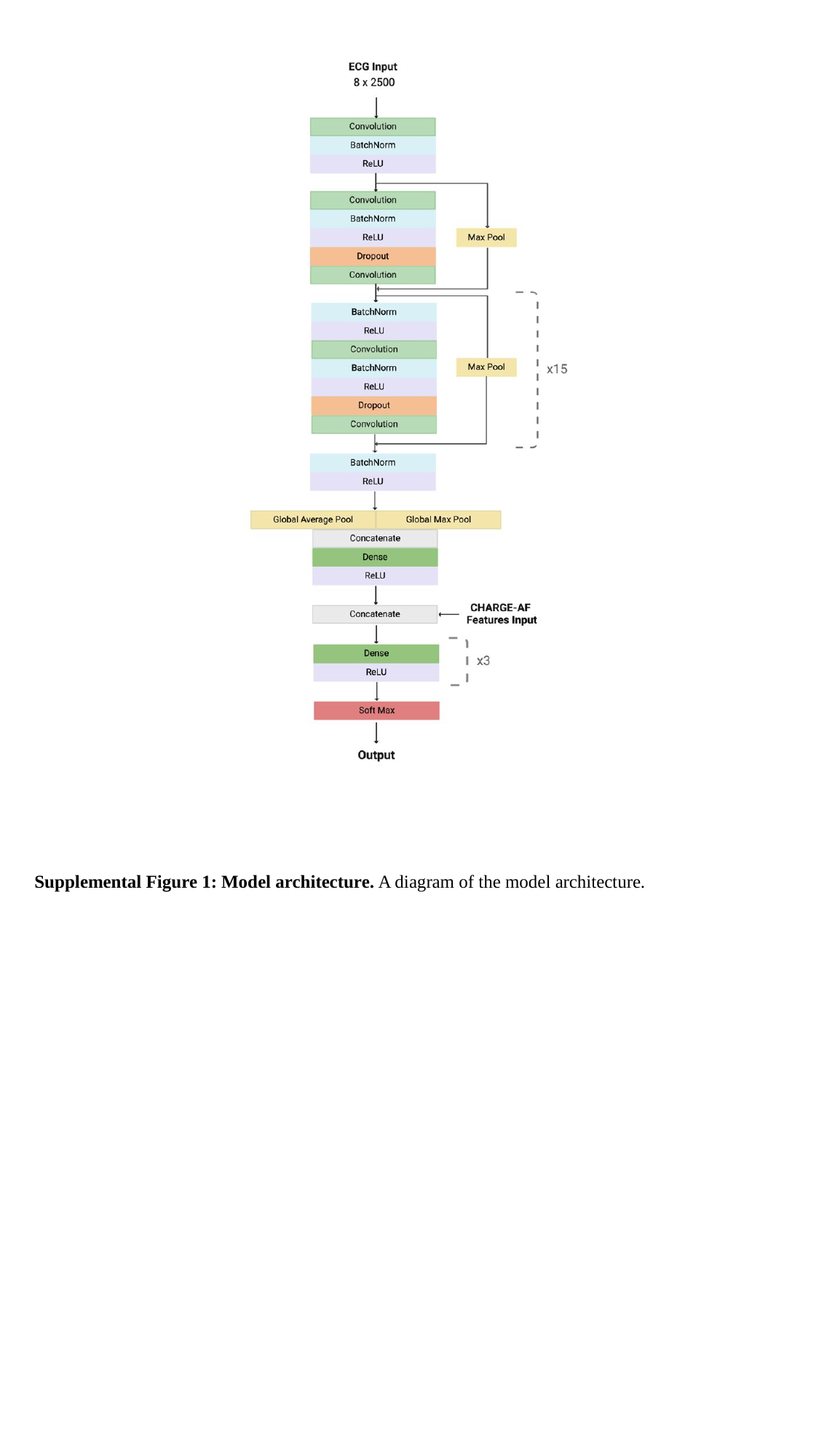

Supplemental Figure 1: Model architecture. A diagram of the model architecture.

## Slide 2
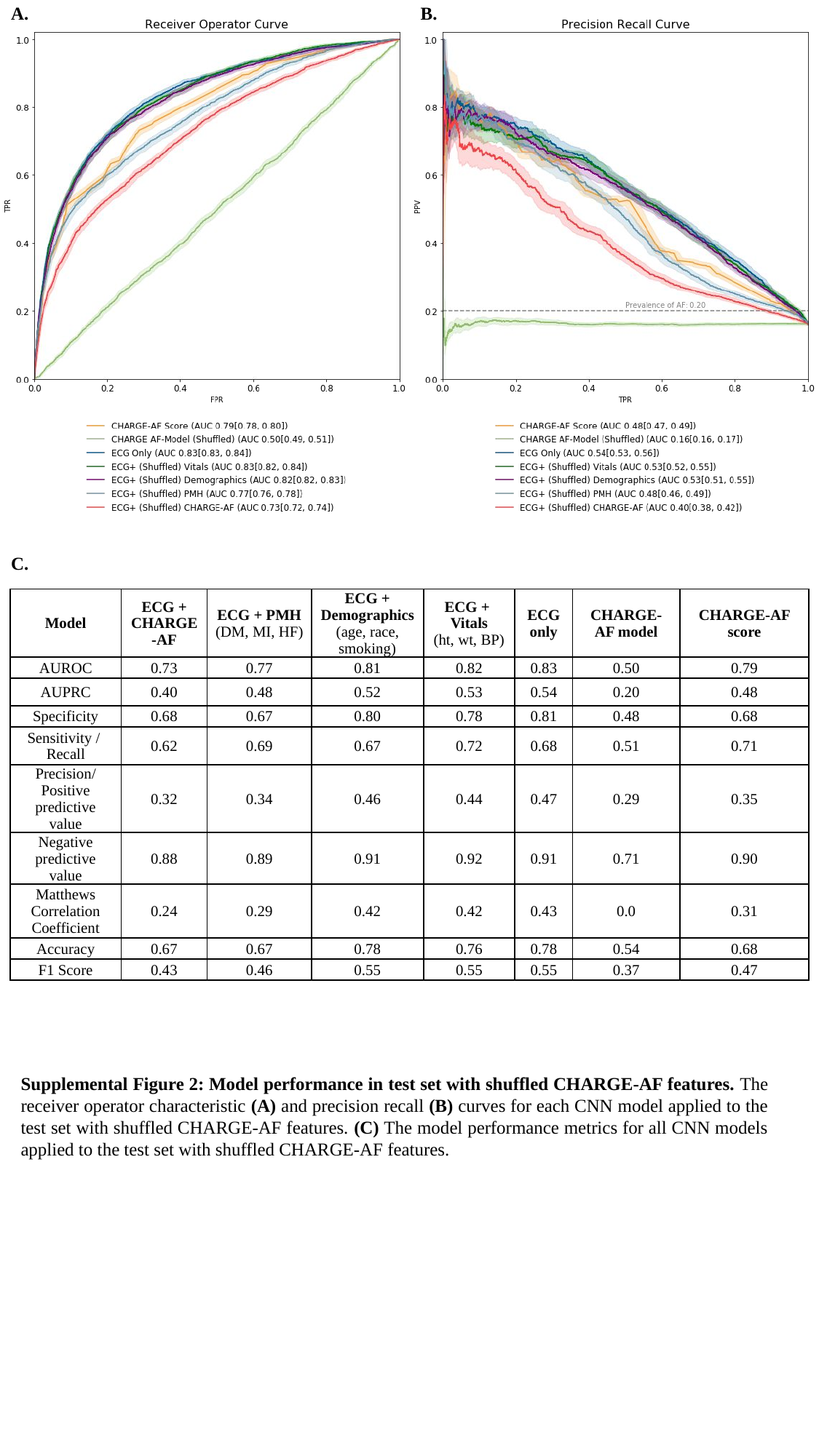

B.
A.
C.
| Model | ECG + CHARGE-AF | ECG + PMH (DM, MI, HF) | ECG + Demographics (age, race, smoking) | ECG + Vitals (ht, wt, BP) | ECG only | CHARGE-AF model | CHARGE-AF score |
| --- | --- | --- | --- | --- | --- | --- | --- |
| AUROC | 0.73 | 0.77 | 0.81 | 0.82 | 0.83 | 0.50 | 0.79 |
| AUPRC | 0.40 | 0.48 | 0.52 | 0.53 | 0.54 | 0.20 | 0.48 |
| Specificity | 0.68 | 0.67 | 0.80 | 0.78 | 0.81 | 0.48 | 0.68 |
| Sensitivity / Recall | 0.62 | 0.69 | 0.67 | 0.72 | 0.68 | 0.51 | 0.71 |
| Precision/ Positive predictive value | 0.32 | 0.34 | 0.46 | 0.44 | 0.47 | 0.29 | 0.35 |
| Negative predictive value | 0.88 | 0.89 | 0.91 | 0.92 | 0.91 | 0.71 | 0.90 |
| Matthews Correlation Coefficient | 0.24 | 0.29 | 0.42 | 0.42 | 0.43 | 0.0 | 0.31 |
| Accuracy | 0.67 | 0.67 | 0.78 | 0.76 | 0.78 | 0.54 | 0.68 |
| F1 Score | 0.43 | 0.46 | 0.55 | 0.55 | 0.55 | 0.37 | 0.47 |
Supplemental Figure 2: Model performance in test set with shuffled CHARGE-AF features. The receiver operator characteristic (A) and precision recall (B) curves for each CNN model applied to the test set with shuffled CHARGE-AF features. (C) The model performance metrics for all CNN models applied to the test set with shuffled CHARGE-AF features.

## Slide 3
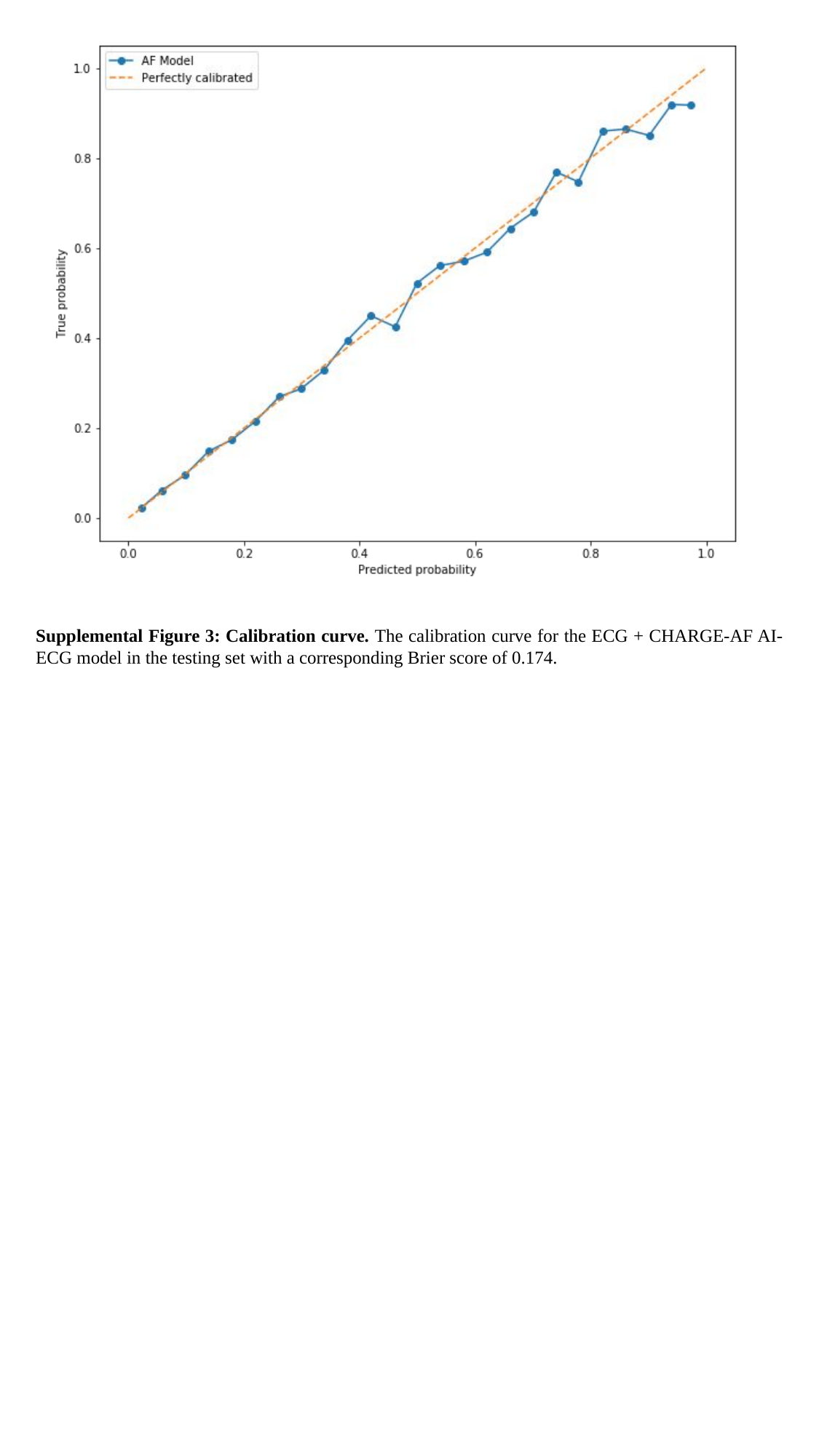

Supplemental Figure 3: Calibration curve. The calibration curve for the ECG + CHARGE-AF AI-ECG model in the testing set with a corresponding Brier score of 0.174.
