# Supplementary material for: Artificial intelligence–enabled sinus electrocardiograms for the detection of paroxysmal atrial fibrillation benchmarked against the CHARGE-AF score
Source: Eur Heart J Digit Health. 2025 Aug 22;6(6):1134–44. doi: 10.1093/ehjdh/ztaf100 (PMC12629645; doi:10.1093/ehjdh/ztaf100)
Supplement: ztaf100_Supplementary_Data [file ztaf100_supplementary_data.zip › Supp tables final 7.10.docx]

| **Datasets:**  ECGs, N (%)  Patients, N (%) | **Main cohort**  157,192 (100%)  76,986 (100%) | **Train**  109,535 (60%)  53,886 (70%) | **Validation**  15,964 (20%)  7,757(10%) | **Test**  31,693 (20%)  15,343(20%) | **p value** | **Missing**  N (% of main cohort) |
| --- | --- | --- | --- | --- | --- | --- |
| **Age,** Median (IQR), y | 64.4 [52.5, 74.4] | 64.4 [52.4, 74.4] | 64.8 [52.6, 74.6] | 64.3 [52.6, 74.4] | 0.261 | 0 (0) |
| **Female sex**, N (%) | 38146 (49.6) | 26779 (49.7) | 3823 (49.3) | 7544 (49.2) | 0.460 | 7 (0.01) |
| **Race and Ethnicity,** N (%) |  |  |  |  | 0.630 | 233 (0.2%) |
| **White** | 47999 (62.5) | 33696 (62.7) | 4832 (62.4) | 9471 (61.9) |  |  |
| **Black** | 9273 (12.1) | 6467 (12.0) | 932 (12.0) | 1874 (12.2) |  |  |
| **Asian** | 5,126 (6.7) | 3563 (6.6) | 524 (6.8) | 1030 (6.7) |  |  |
| **Hispanic or Latino** | 1559 (2.0) | 1081 (2.0) | 154 (2.0) | 324 (2.1) |  |  |
| **American Indian or Alaska Native** | 265 (0.3) | 187 (0.3) | 21 (0.3) | 57 (0.4) |  |  |
| **Native Hawaiian or Pacific Islander** | 159 (0.2) | 104 (0.2) | 18 (0.2) | 37 (0.2) |  |  |
| **Middle Eastern or North African** | 62 (0.1) | 47 (0.1) | 1 (0.0) | 14 (0.1) |  |  |
| **Other** | 12388 (16.1) | 8624 (16.0) | 1259 (16.3) | 2505 (16.4) |  |  |
|  |  |  |  |  |  |  |
| **BMI,** Median (IQR), kg/m^2^ | 27.4 [23.7, 31.9] | 27.4 [23.7, 31.9] | 27.3 [23.7, 31.8] | 27.4 [23.8, 31.9] | 0.780 | 0 (0) |
| **Smoking**, N (%) |  |  |  |  | 0.596 | 332 (0.8%) |
| **Current** | 5927 (7.7) | 4138 (7.7) | 582 (7.5) | 1207 (7.9) |  |  |
| **Former** | 27619 (36.0) | 19415 (36.2) | 2776 (36.0) | 5428 (35.6) |  |  |
| **Never** | 43108 (56.2) | 30115 (56.1) | 4360 (56.5) | 8633 (56.5) |  |  |
| **Comorbidities**, N (%) |  |  |  |  |  | 0 (0) |
| **Diabetes** | 12043 (15.6) | 8367 (15.5) | 1239 (16.0) | 2437 (15.9) | 0.395 |  |
| **Heart failure** | 11376 (14.8) | 7971 (14.8) | 1162 (15.0) | 2243 (14.6) | 0.753 |  |
| **Hypertension** | 18449 (24.0) | 12913 (24.0) | 1854 (23.9) | 3682 (24.0) | 0.987 |  |
| **Myocardial Infarction** | 9159 (11.9) | 9159 (11.9) | 946 (12.2) | 1811 (11.8) | 0.670 |  |
| **Vital signs,** Median (IQR), mm Hg |  |  |  |  |  | 0 (0) |
| **Systolic blood pressure** | 125 [113, 139] | 125 (113, 138) | 125 (113, 139) | 125 (113, 139) | 0.493 |  |
| **Diastolic blood pressure** | 73 [65, 80] | 73 (65, 80) | 73 (65, 80) | 73 (65, 80) | 0.329 |  |
| **Medications**, N (%) |  |  |  |  |  | 0 (0) |
| **Anti-hypertensives** | 10270 (13.3) | 7220 (13.4) | 1028 (13.3) | 2022 (13.2) | 0.436 |  |
| **CHARGE-AF,** Median (IQR) | 0.015 [0.004, 0.046] | 0.015 [0.004, 0.046] | 0.015 [0.004, 0.047] | 0.015 [0.004, 0.046] | 0.420 | 0 (0) |
| **Atrial Fibrillation within 90 days** |  |  |  |  |  |  |
| ECGs, N (%) | 45206 (28.8) | 8840 (27.9) | 31737 (29.0) | 4629 (29.0) | 0.001 | 0 (0) |
| Patients, N (%) | 16095 (20.9) | 11343 (21.0) | 1688 (21.8) | 3064 (20.0) | 0.002 |  |

**Supplemental Table 1: Baseline characteristics.**

A breakdown of the demographic characteristics, comorbidities, vital signs, CHARGE-AF score and AF proportion for the entire cohort, as well as the train, validation, test and held-out test datasets.

Abbreviations: BMI = body mass index, ECG = electrocardiogram, IQR = interquartile range, N = count, y = years.

| ECGs, N (%)  Patients, N (%) | **Local External Validation Cohort**  5,488 (100%)  4,215 (100%) |
| --- | --- |
| **Age,** Median (IQR), y | 66.3 [55.5, 76.3] |
| **Female sex**, N (%) | 2242 (53.2) |
| **Race and Ethnicity,** N (%) |  |
| **White** | 3170 (75.6) |
| **Black** | 305 (7.3) |
| **Asian** | 134 (3.2) |
| **Hispanic or Latino** | 11 (0.3) |
| **American or Alaska Native** | 3 (0.07) |
| **Native Hawaiian or Pacific Islander** | 1 (0.02) |
| **Middle Eastern or North African** | 3 (0.07) |
| **Other** | 565 (13.4) |
|  |  |
| **BMI,** Median (IQR), kg/m^2^ | 27.8 [24.4, 31.9] |
| **Smoking**, N (%) |  |
| **Current** | 224 (5.3) |
| **Former** | 1618 (38.4) |
| **Never** | 2373 (56.3) |
| **Comorbidities**, N (%) |  |
| **Diabetes** | 344 (8.2) |
| **Heart failure** | 249 (5.9) |
| **Hypertension** | 759 (18.0) |
| **Myocardial Infarction** | 68 (1.6) |
| **Vital signs,** Median (IQR), mm Hg |  |
| **Systolic blood pressure** | 124 [118,134] |
| **Diastolic blood pressure** | 74 [70, 80] |
| **Medications**, N (%) |  |
| **Anti-hypertensives** | 378 (9.0) |
| **CHARGE-AF,** Median (IQR) | 0.017 [0.005, 0.046] |
| **Atrial Fibrillation within 90 days** |  |
| ECGs, N (%) | 871 (15.9) |
| Patients, N (%) | 535 (12.7) |

**Supplemental Table 2: US (NYU Long Island) External validation cohort.**

A breakdown of the demographic characteristics, comorbidities, vital signs, CHARGE-AF score and AF proportion for the external validation cohort derived from NYU Long Island.

Abbreviations: BMI = body mass index, ECG = electrocardiogram, IQR = interquartile range, N = count, y = years

| ECGs, N (%)  Patients, N (%) | **European External Validation Cohort**  306 (100%)  306 (100%) |
| --- | --- |
| **Age,** Median (IQR), y | 57 [42, 70] |
| **Female sex**, N (%) | 203 (66) |
| **Race and Ethnicity,** N (%) |  |
| **White** | 304 (99) |
| **Asian** | 2 (1) |
|  |  |
| **BMI,** Median (IQR), kg/m^2^ | 26.8 [23.2, 30.5] |
| **Smoking**, N (%) | 94 (31) |
| **Comorbidities**, N (%) |  |
| **Diabetes** | 39 (12.7) |
| **Heart failure** | 21 (6.9) |
| **Hypertension** | 106 (34.6) |
| **Myocardial Infarction** | 9 (2.9) |
| **Vital signs,** Median (IQR), mm Hg |  |
| **Systolic blood pressure** | 128 [115,140] |
| **Diastolic blood pressure** | 77.5 [70, 80] |
| **CHARGE-AF,** Median (IQR) | 0.014 [0.002, 0.056] |
| **Atrial Fibrillation**, N (%) | 128 (41) |

**Supplemental Table 3: European (Greece) External validation cohort.**

A breakdown of the demographic characteristics, comorbidities, vital signs, CHARGE-AF score and AF proportion for the external validation cohort derived from NYU Long Island.

Abbreviations: BMI = body mass index, ECG = electrocardiogram, IQR = interquartile range, N = count, y = years

| **Item #** | **Category/Section** | **Explanation** | **Page #** |
| --- | --- | --- | --- |
|  | **TITLE** |  |  |
|  | ***Title*** | Include clear terms to identify the study as using artificial intelligence, machine learning or other specific terms | Title page: **“**Artificial intelligence-enabled sinus electrocardiograms for the detection of paroxysmal atrial fibrillation benchmarked against the CHARGE-AF score” |
|  | **INTRODUCTION** |  |  |
| 1 | **Intended clinical use** | Clearly describe the intended use and where in clinical workflow the model can be used and the objective of the study | Page 2: “To inform targeted screening, neural networks utilizing the sinus ECG signal as their primary input (AI-ECG) have been developed for the detection of paroxysmal AF…” |
| 2 | **Clinical benefit** | Added benefit of AI compared to standard clinical care (gold standard) | Page 2: “However, there are conflicting reports on the additive AF predictive value of such AI-ECG models against established clinical risk scores like the CHARGE-AF^13^ score, illustrating either equivalent^10,11^ or only marginally greater^12^ predictive power. We report an ECG-based convolutional neural network (CNN) for the detection of incident AF illustrating its superior performance and clinical utility against the CHARGE-AF score and externally validating it in both local suburban outpatient practices and European tertiary referral hospitals.” |
|  | **METHODS** |  |  |
| 3 | **Data Collection** | Describe how data was collected | Page 3: “We identified all 12-lead ECGs obtained within the New York University (NYU) Langone Health system between January 1, 2012 and January 1, 2022…” |
| 4 | **Source (of data)** | Describe the study design or source of input data and how it was acquired | Page 4: “Encounter data were collected from the NYU Langone Health EHR (Epic Systems, Verona, WI) and ECGs were retrieved from MUSE as XML files (GE Healthcare, Chicago, IL).” |
| 5 | **Development data set (model training data set)** | Describe the data set | Page 3: “…The resulting study cohort of NSR ECGs belonging to patients with and without a history of AF, was split into training, validation, and test sets in a 7:1:2 ratio (Fig. 1B) and their characteristics are listed in Supplemental Table 1...” |
| 6 | **Participants** | Describe the participants in the data sets, including eligibility criteria (inclusion and exclusion criteria). | Page 3: “The cohort included two groups of patients based on the presence or absence of a documented history of AF ascertained by ECG labels generated by MUSE (GE Healthcare, Chicago, IL). For patients with no AF ECG recorded, all NSR ECGs were identified and included. For patients with at least one AF ECG recorded, all NSR ECGs obtained within ±90 days of an AF ECG were identified and included. If multiple AF ECGs were obtained within 180 days of each other, the earliest (index) AF ECG occurrence was used to identify NSR ECGs for inclusion (Fig. 1A). Patients who had no record of an AF ECG in the electronic health record (EHR), but otherwise had a documented history of an AF diagnosis were excluded from the study population.” |
| 7 | **Comparator** | Provide clear definition of how the gold standard was collected.  Clearly describe the gold standard and ground truth including limitations. | Page 4: “The CHARGE-AF score was calculated using clinical features that preceded each ECG acquisition date. If CHARGE-AF features were missing from the EHR, then these cases were excluded. The CHARGE-AF score was calculated as previously described^13^ including the following features: past medical history (diabetes, myocardial infarction, heart failure), demographics (age, race, current smoking), vitals (height, weight, systolic/diastolic blood pressure) and use of antihypertensive medication.” |
| 8 | **Validation data set** | Describe the validation data set, in particular defining the data set split. | Page 3: “The ECGs obtained at NYU Long Island served as an external validation cohort and were not included in the main cohort (Fig. 1B, Supplemental Table 2). A second external validation cohort (Supplemental Table 3) was derived from Miteral Hospital, a private hospital located in Athens, Greece and the Ippokrateio General Hospital, a public hospital affiliated with the Aristotle University of Thessaloniki (AUTH), also located in Greece. This European external validation cohort included NSR ECGs at any time point in relation to an AF episode.” |
| 9 | **Sample Size** | Explain how the study size was arrived at. | Page 3: “The resulting study cohort of NSR ECGs belonging to patients with and without a history of AF, was split into training, validation, and test sets in a 7:1:2 ratio (**Fig. 1B**) and their characteristics are listed in Supplemental Table 1.” |
| 10 | **Outcome** | Clearly define standardized and reproducible outcome of clinical relevance. | Page 4: “A total of five models were developed as described below to detect incident AF from NSR ECGs” |
| 11 | **Data type (source)** | Clearly describe the data type for the study, including pre-processing | Page 4: “Encounter data were collected from the NYU Langone Health EHR (Epic Systems, Verona, WI) and ECGs were retrieved from MUSE as XML files (GE Healthcare, Chicago, IL).” |
| 12 | **Data Preparation** | Input data handling, data augmentation and selection prior to analysis by the AI system. | Page 4: “A total of five models were developed as described below to detect incident AF from NSR ECGs: ECG + CHARGE-AF, ECG + past medical history (PMH), ECG + demographics, ECG + vitals and ECG only. As suggested by their names, the models differed in the type of inputs used for model development, which included the NSR ECG time series and tabular data corresponding to all or subsets (as described above) of the CHARGE-AF score’s features. They were compared with the CHARGE AF score and a CHARGE-AF model that used the features as inputs into a CNN. Whenever more than one NSR ECG was available and eligible for inclusion as determined by the study design (Fig. 1A), then the time series for all were utilized as model inputs during training.” |
| 13 | **Balanced groups** | Clearly state how groups were balanced | N/A |
| 14 | **Data issues (missingness / poor data / duplication / outliers)** | Describe how handling of data of poor quality/noise/missing data was performed | Page 4: “If CHARGE-AF features were missing from the EHR, then these cases were excluded.” |
| 15 | **Feature extraction / selection / reduction** | If features are used, feature selection should be described including by whom features were extracted. | Page 4: “The CHARGE-AF score was calculated as previously described13 including the following features: past medical history (diabetes, myocardial infarction, heart failure), demographics (age, race, current smoking), vitals (height, weight, systolic/diastolic blood pressure) and use of antihypertensive medication…” |
|  | **REGULATORY** |  |  |
| 16 | **Legal framework** | Clearly state if the software has been approved by legal authorities, e.g. Certificate of conformity (EU) or FDA approval or other, and add further details, where appropriate (e.g. risk class). | N/A: the very development of the software is being described in the present manuscript and is not already approved |
| 17 | **Explainability** | Is the AI model explainable on the patient level or on a population level. | N/A: convolutional neural network, which to date is largely a “black box” model |
| 18 | **Ethical approval** | Provide information on ethical approval of the study. | Pages 3-4: “Both the NYU Langone Health Institutional Review Board and Independent Ethics Committee for each hospital in Greece, approved the study protocol and waived the need for informed consent. The study complied with the principles of the Declaration of Helsinki.” |
| 19 | **Fairness** | Describe inclusion of relevant groups in the dataset | Breakdowns by age, sex and race included in:  - Supplemental Table 1  - Figure 2D  - Figure 3D |
|  | **OPEN SCIENCE** |  |  |
| 20 | **Data availability/ Code sharing** | Is the data available on a public website? Is the code available? | The datasets used and/or analyzed during the current study are available from the corresponding author on reasonable request. |
| 21 | **Trial registration** | Clearly state where the trial is registered. | N/A |
|  | **RESULTS** |  |  |
| 22 | **Participants** | Baseline demographics | Pages 6-9 and Supplemental Tables 1-3 |
| 23 | **Training performance** | Provide results from the training data set | Page 7 and Figure 2 |
| 24 | **Internal validation** | The results from the testing data set | Page 7 and Figures 2 |
| 25 | **External validation** | The results from the external validation data set | Pages 8-9 and Figures 3-4 |
| 26 | **Model performance Internal and external validation** | Choose appropriate metric selection for reporting | AUC, AUPRC, sensitivity, specificity, precision/positive predictive value, negative predictive value, Matthew’s correlation coefficient, Accuracy, F1 score (Figures 2-4) |
| 27 | **Performance errors** | Analysis of performance errors and how they were identified | Pages 7-8 and Supplemental Figure 2 |
| 28 | **Performance compared to classic statistical methods** | What did the model add? | Comparison against the CHARGE-AF score, Pages 6-9 and Figures 2-4 |
| 29 | **Generalizability** | Describe the population (internal and external validation data) | Pages 6-9, Supplemental Tables 1-3 |
|  | **CONCLUSION** |  |  |
|  | **Conclusion** | Is the conclusion supported by the dataset? | Pages 10-14, analyzed in Discussion section |

**Supplemental Table 4: EHRA AI Checklist**

The EHRA AI checklist for reporting, reading and understanding AI studies in clinical EP.

Abbreviations: AI = artificial intelligence, EHRA = European Heart Rhythm Association, EP = electrophysiology.
